# Physiological Sleep Propensity Might Be Unaffected by Significant Variations in Self-Reported Well-Being, Activity, and Mood

**DOI:** 10.1155/2015/532831

**Published:** 2015-07-30

**Authors:** Arcady A. Putilov

**Affiliations:** Research Institute for Molecular Biology and Biophysics, 2 Timakova Street, Novosibirsk 630117, Russia

## Abstract

*Background and Objective*. Depressive state is often associated with such physical symptoms as general weakness, fatigue, tiredness, slowness, reduced activity, low energy, and sleepiness. The involvement of the sleep-wake regulating mechanisms has been proposed as one of the plausible explanations of this association. Both physical depressive symptoms and increased physiological sleep propensity can result from disordered and insufficient sleep. In order to avoid the influence of disordered and insufficient sleep, daytime and nighttime sleepiness were tested in winter depression characterized by normal night sleep duration and architecture. *Materials and Methods*. A total sample consisted of 6 healthy controls and 9 patients suffered from depression in the previous winter season. Sleep latency was determined across 5 daytime and 4 nighttime 20-min attempts to nap in summer as well as in winter before and after a week of 2-hour evening treatment with bright light. *Results and Conclusions*. Patients self-reported abnormally lowered well-being, activity, and mood only in winter before the treatment. Physiological sleep propensity was neither abnormal nor linked to significant changes in well-being, activity, and mood following the treatment and change in season. It seems unlikely that the mechanisms regulating the sleep-wake cycle contributed to the development of the physical depressive symptoms.

## 1. Introduction

Depression is often associated with such subjective physical complaints as general weakness, fatigue, tiredness, slowness, reduced activity, low energy, and sleepiness [1]. Epidemiological and clinical studies have pointed out that this association might be very strong [2–9], rather complex and bidirectional [10]. Its biological basis has not been fully established [11]. Among several plausible explanations, the involvement of the sleep-wake regulating mechanisms has been proposed [12–14]. However, objective evidence has not been yet provided to support the view that depression is associated with abnormal (e.g., either increased or decreased) physiological sleep propensity. At least, one can draw this conclusion from the results of several independent studies [15–18] applying objective tools of direct measurement of the levels of daytime and nighttime sleepiness, such as the Multiple Sleep Latency Test (MSLT).

Both the physical depressive complaints and increased physiological sleep propensity can result from disordered and insufficient sleep. The contribution of the disordered and insufficient sleep can be avoided in the study of those mildly or moderately depressed conditions that are not obligatorily associated with disruption of nighttime sleep and hyposomnia. Such a study can shed light on the involvement of sleep-wake regulating mechanisms in the development and remission of depressive state. One of such conditions is winter depression or seasonal affective disorder of winter type. Frequent occurrence of hypersomnia and hyperphagia was stressed in its initial description [19]. Moreover, our earlier published polysomnographic research of night sleep in winter depression revealed neither its abnormality in terms of duration and architecture nor its significant effects of the change in season and treatment with bright light on the vast majority of objective characteristics of night sleep [20].

However, it is unknown whether physiological sleep propensity also remains unaffected in this condition. Therefore, the major aims of this report were (i) to test objectively the levels of daytime and nighttime sleepiness in winter depression during remission and depressed episode and (ii) to examine strength of relationship of objectively measured sleepiness with lowered well-being, activity, and mood in depressed condition.

## 2. Materials and Methods

The experimental protocol was approved by the Ethics Committee of the Siberian Branch of the Russian Academy of Medical Sciences. All 15 study participants gave written informed consent. The participants were unmedicated, in good general health, and free of any sleep disorders and were not engaged in shift work or long distance travel in the previous 2 months. Controls were also free from any types of mood and psychosomatic disorders. The criteria of Rosenthal et al. [19] were applied for the diagnosis of winter depression. They, however, were more stringent in that a history of complete summer remission was required (see [21, 22] for details). A total sample included 9 women with winter depression. Their depressive episode was clinically diagnosed during the previous study conducted in the winter season. The ages of patients (mean ± SD) were 34.2 ± 13.0 (range from 17 to 56 years old). The ages of 6 women from the control group were in the range from 22 to 55 (40.5 ± 9.7 years old).

Six women from each group were first studied in summer. Then, five women from each group were restudied in winter, and the patient group was enlarged by adding 3 depressed women with winter depression. Of these 13 participants of the winter study, 7 patients and 4 controls were restudied after a week of 2-hour evening treatment with bright light (2500 lux at the eyes' level). The study was terminated at this stage because the preliminary analysis of its results revealed neither abnormality in the levels of sleepiness in the patient group nor indication of relationship of objectively measured sleepiness with lowered well-being, activity, and mood in depressed condition.

Current clinical symptoms were rated with the 21-item Hamilton Rating Scale for Depression (HRSD) [23]. Additionally, the depressive symptoms were self-rated with the 29-item Structured Interview Guide for the Hamilton Depression Rating Scale: Seasonal Affective Disorder (SIGH). The SIGH includes the HRSD and 8-item Addendum concerning atypical depressive symptoms, such as hypersomnia, carbohydrate craving, increased appetite, increased eating, weight gain, fatigue, type B (inversed) diurnal variation, and social withdrawal [24].

After an adaptation night, polysomnographic records were obtained during normally scheduled night sleep (23:00–8:00) and, thereafter, during 9 20-min sleep latency tests in daytime (10:00, 12:00, 14:00, 16:00, and 18:00) and nighttime (23:00, 1:00, 3:00, and 5:00). In the course of any of these recordings the participants were lying in bed in the darkened room of the sleep laboratory and were asked to try to fall asleep and sleep until hearing the awakening signal from the nursing staff.

In order to self-assess subjective state before and after baseline night sleep and before each of 9 20-min sleep attempts, the participants filled the 30-item WAM-Test consisting of 3 10-item subscales named “well-being,” “activity,” and “mood” [25]. Each subscale includes 10 word pairs with a 7-point response scale printed between each pair of words. The following words exemplify these 3 subscales:

*Well-Being*

 
1. Good health  3210123  Bad health
 
2. Feel strong  3210123  Feel weak
 
7. Able to work  3210123  Broken

*Activity*

 
3. Passive   3210123  Active
 
4. Sedentary 3210123  Agile
 
9. Sluggish  3210123  Quick

*Mood*

 
5. Gleeful    3210123  Sad
 
6. Good mood  3210123  Bad mood
 
11. Happy    3210123  Fortuneless

After transforming each of the response scales “3210123” into “1234567” or “7654321,” a summing score for a subscale varies between 10 and 70. Low, middle, and high levels of subjective state can be distinguished by assigning scores to the intervals <30, 30–50, and >50, respectively [25].

Polysomnographic data were collected via an 8-channel Medicor polygraph (EEG8S, Micromed, Hungary). The sleep recordings were performed using a standard monitoring montage including five EEG channels, two electrooculogram channels, and one chin electromyogram channel. The reported data were mostly taken from Cz-A1 derivation of the International Ten-Twenty System of Electrode Placement (i.e., vertex of the head versus left mastoid). To fix the electrodes, Ten20 conductive paste was used (Nicolet Biomedical, Madison, Wisconsin, USA). Sleep stages were visually scored by two independent judges. The epochs with discrepant scores were reexamined by both judges together to produce consensus scores. Each epoch of the polysomnographic record was categorized in accord with the standard criteria [26, 27] as wake, stage 1, stage 2, and stages 3 and 4 (slow wave sleep) of non-REM sleep, REM sleep, or movement.

All statistical analyses were performed with the Statistical Package for the Social Sciences (SPSS) ver. 20.0 (IBM, Armonk, NY, USA). Student's* t*-test was applied to evaluate the differences between patient and control groups on subjective state ratings (Table 1) and on objective evaluations of baseline night sleep and napping attempts (Table 2). Additionally, the effects of treatment on repeated self-ratings and repeated objective measures were tested separately on daytime and nighttime intervals with 3-way repeated measure ANOVAs (rANOVAs). The within-subjects factors were “Time of day” (from 10:00 to 18:00 for the experimental day, and from 23:00 to 5:00 for the baseline and experimental nights) and “Condition” (before versus after the bright light treatment). The between-subjects factor was “Group” (patients versus controls). To control for the type 1 error associated with violation of the assumption of sphericity, all *p* values were based on Huynh-Feldt's correction of the degrees of freedom, but the original degrees of freedom are reported in the text. Prior to applying these analyses, normality of distribution was tested for each variable by means of the exploratory analysis. The hypothesis of normality (Kolmogorov-Smirnov Z-criterion) was always supported (*p* > 0.05).

## 3. Results

The self-reported daytime scores on the subscales of well-being, activity, and mood were high in the study participants from the control group at any of the three occasions. As expected, the participants form the patient group providing either high or almost high self-scorings of well-being, activity, and mood in summer and in winter after the treatment. In contrast, they were found to be either low or somewhere on the border between low and middle scores in the winter season before the treatment (Table 1). The significant differences between the groups and conditions persisted in the nighttime hours, and, as expected, each score was found to be lowered at night as compared to the corresponding score for daytime hours (Table 1).

Such pattern of the differences between the groups on self-scorings of well-being, activity, and mood was confirmed by rANOVAs. For instance, the analysis of daytime scores obtained by averaging over 3 subscales yielded significant main effect of the factor “Group” as well as significant interaction between this factor and factor “Condition”: *F*(*N* = 11, df = 1) = 10.4, *p* = 0.010 and *F*(*N* = 11, df = 1) = 6.0, *p* = 0.037, respectively. This interaction indicated dramatic increase of score in the patient group in response to the treatment (see the first line in Table 1).

**Figure 1:**
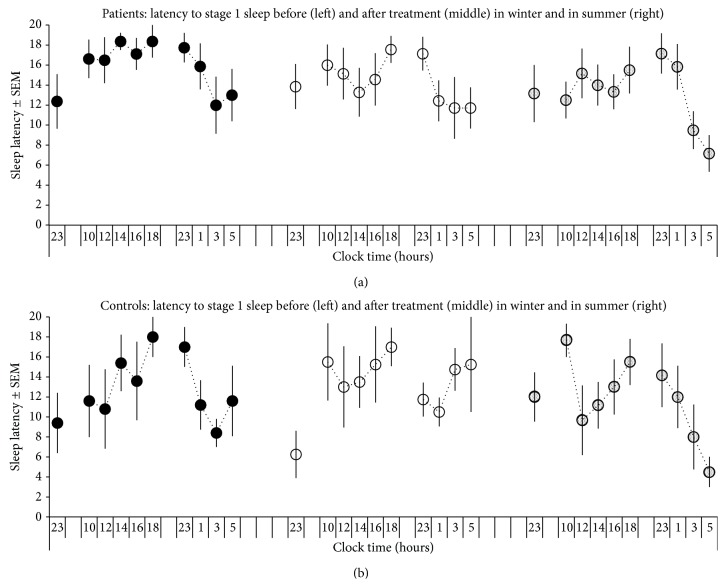
Time course of sleep latency in patients and controls. Sleep latency was tested 10 times on three occasions (in summer and in winter before and after treatment), during the first 20 min of baseline night sleep and during 5 daytime and 4 nighttime 20-min attempts to nap. See also notes to Table 1 and the results of comparison of patient and control groups in Table 2.

The opposite pattern of interaction between factors “Group” and “Condition” was revealed by rANOVA of daytime sleep latency: *F*(*N* = 11, df = 1) = 5.6, *p* = 0.043. As can be seen in Figure 1, sleep latency showed a tendency to decrease in patients and increase in controls due to the treatment. Unlike subjective scorings, sleep latency in winter before the treatment was not lowered in patients and did not show significant difference from that of controls (Table 2).

Moreover, the absence of significant changes in sleep latency was confirmed by comparison of mean latencies that were obtained for each participant by averaging over 10 sleep attempts. For instance, none of patients showed lowered mean sleep latency in winter before treatment as compared to that in summer season or in winter after the treatment.

Finally, architecture of baseline night sleep in patients did not differ much from architecture observed in the control group. Instead, participants from the patient group slept even longer than controls in both seasons (Table 2).

## 4. Discussion

The biological basis of the association of mood disorders with such physical symptoms as general weakness, fatigue, tiredness, slowness, reduced activity, low energy, and sleepiness is poorly understood. Particularly, it remains unknown whether the chronophysiological mechanisms, such as the sleep-wake regulating processes, can contribute to the development of depression and to a beneficial response to an antidepressant treatment. The present study results failed to provide evidence for such contribution. They indicate that the physiological sleep propensity in the patient group was normal and its changes were unrelated to changes in the subjectively reported well-being, activity, and mood. These results imply that wintertime self-scorings of well-being, activity, and mood can be lowered in depressed individuals despite the absence of any significant differences between them and healthy people in objectively measured sleep latency.

However, the limitations of the present analysis include small sample sizes and the absence of severe depressed participants in the patients group. Future studies lacking these limitations might provide a deeper insight into the relationship between depressed mood and sleep-promoting processes.

The treatment and summer conditions were associated with normalization of self-scoring in the patient group. This result is in line with the findings of our earlier studies of sleep-wake patterns in bigger samples of patients with winter depression and healthy controls. In one of these studies [20, 28, 29] we similarly found that patients self-scored lower than controls their levels of well-being, activity, and mood in winter before light treatment in the morning and afternoon hours, but not after such a treatment. Moreover, the scores were found to be high and identical to those reported by the controls during retesting of subsamples of the participants of winter study in the following summer season. In another experimental investigation [30, 31], we demonstrated significant differences between patients and controls on wintertime levels of sleepiness self-assessed with the Karolinska Sleepiness Scale (KSS) and levels of energy self-reported with a visual analog scale.

It is likely that such strong association of health status with activity and sleepiness levels is a very general feature of introindividual variation in subjective states. At least, this association was reported not only for unhealthy but also for practically healthy individuals. For instance, our results resemble the findings reported by Åkerstedt et al. [32]. These findings suggested that worsening of self-perceived health status was the strongest predictor of the increase in KSS self-scorings, whereas the markers of disturbed sleep, such as shorter preceding sleep duration, earlier time of rising, and lower-rated sleep quality, were among less strong albeit yet significant predictors.

## 5. Conclusions

Physiological sleep propensity was neither abnormal in wintertime as compared to summertime nor linked to the changes in well-being, activity, and mood in winter depression with normal sleep architecture and duration. Therefore, it seems unlikely that the mechanisms regulating the sleep-wake cycle can contribute to the development of the physical complaints of reduced activity and sleepiness in mildly or moderately depressed patients.

## Figures and Tables

**Table 1:** Results of *t*-test aimed at comparison of assessments of subjective state.

Season	Winter either before or after treatment				Summer	
Condition	Before		After		Remission	
Group	Patients	Controls	Patients	Controls	Patients	Controls
Daytime WAM (well-being-activity-mood) scorings						
30-item averaged	36.6 ± 10.3^*∗∗*^	54.3 ± 6.4	51.4 ± 5.8	56.9 ± 5.8	47.2 ± 10.2	53.6 ± 8.5
10-item well-being	37.8 ± 11.5^*∗∗*^	57.2 ± 5.4	52.5 ± 5.4	59.4 ± 5.4	48.2 ± 12.1	55.7 ± 9.1
10-item activity	32.0 ± 10.2^*∗∗*^	52.2 ± 8.4	48.3 ± 6.2^*∗*^	57.8 ± 4.9	42.3 ± 11.0	51.7 ± 9.1
10-item mood	39.9 ± 9.9^*∗*^	53.4 ± 6.1	53.4 ± 6.7	53.5 ± 8.6	51.1 ± 6.9	53.6 ± 9.6

Nighttime WAM scorings						
30-item averaged	33.3 ± 8.5^*∗*^	46.5 ± 7.3	37.0 ± 7.2^*∗*^	48.8 ± 8.1	43.3 ± 6.7	43.6 ± 10.2
10-item well-being	32.4 ± 8.8^*∗*^	46.1 ± 11.1	34.2 ± 6.6^*∗*^	48.4 ± 11.6	44.2 ± 8.3	46.1 ± 11.3
10-item activity	29.3 ± 8.7^*∗*^	42.4 ± 9.2	33.1 ± 6.9^*∗*^	46.4 ± 6.7	42.9 ± 6.2	42.4 ± 10.3
10-item mood	38.3 ± 8.9^*∗*^	50.9 ± 5.6	43.7 ± 10.1	51.6 ± 6.9	42.9 ± 6.2	42.4 ± 10.3

Depression scorings						
21-item HRSD	16.2 ± 4.7^*∗∗∗*^	2.6 ± 3.8	6.7 ± 2.4^*∗*^	3.0 ± 1.2	7.8 ± 7.1	2.3 ± 3.2
29-item SIGH	30.9 ± 8.1^*∗∗∗*^	5.6 ± 8.2	10.0 ± 3.7^*∗∗*^	3.5 ± 1.9	11.8 ± 9.7	3.3 ± 3.9
8-item Addendum	12.9 ± 3.5^*∗∗∗*^	1.0 ± 1.2	3.1 ± 1.5^*∗∗*^	0.0 ± 0.0	4.0 ± 2.9	1.0 ± 0.8

Notes. WAM: self-scoring on the 30-item WAM Test obtained by averaging over scores provided in daytime and nighttime hours (10:00, 12:00, 14:00, 16:00, and 18:00 and 23:00, 1:00, 3:00, and 5:00, resp.); averaged: mean score obtained by further averaging over 3 10-item self-scorings. HRSD: depression score rated with the 21-item Hamilton Rating Scale for Depression; SIGH: self-scoring on the 29-item Structured Interview Guide for the Hamilton Depression Rating Scale: Seasonal Affective Disorder; Addendum: self-scoring on the Addendum items concerning atypical depressive symptoms (see Materials and Methods). Level of significance for difference between the patient and control groups: ^*∗∗∗*^(*p* < 0.001), ^*∗∗*^(*p* < 0.01), and ^*∗*^(*p* < 0.05).

**Table 2:** Results of *t*-test aimed on comparison of objective measures of sleepiness and sleep.

Season	Winter either before or after treatment				Summer	
Condition	Before		After		Remission	
Group	Patients	Controls	Patients	Controls	Patients	Controls
Latency to stage 1 sleep (SL), min, on 20-min interval of napping attempt and baseline sleep						
Daytime	17.4 ± 3.9	13.9 ± 5.3	15.3 ± 4.2	14.9 ± 4.8	14.1 ± 2.6	13.4 ± 4.7
Nighttime	14.2 ± 5.5	11.5 ± 4.0	13.4 ± 4.9	11.7 ± 4.0	12.6 ± 4.1	10.1 ± 5.4
Baseline	12.4 ± 7.7	9.4 ± 6.7	13.9 ± 6.0	6.3 ± 4.7	6.0 ± 5.9	2.8 ± 3.2

Baseline sleep						
TST, min	483.5 ± 50.3^*∗*^	401.8 ± 68.0	469.9 ± 37.7	437.0 ± 36.5	478.8 ± 42.3^*∗*^	423.7 ± 47.6
SL, min	21.0 ± 23.3	9.4 ± 6.7	23.0 ± 17.5^*∗*^	6.3 ± 4.7	19.2 ± 17.4	12.0 ± 6.0
Wake, min	14.3 ± 5.4	21.6 ± 10.8	11.0 ± 6.6	8.5 ± 6.5	11.3 ± 2.9	36.2 ± 25.5
Stage 1, %	5.2 ± 1.4	7.2 ± 2.6	5.9 ± 1.6^*∗*^	3.6 ± 1.4	7.5 ± 3.8	5.8 ± 1.2
Stage 2, %	64.9 ± 6.0	60.9 ± 6.7	57.7 ± 6.1	64.5 ± 2.4	56.9 ± 9.5	59.9 ± 9.1
SWS, %	9.7 ± 4.9	14.8 ± 4.4	12.8 ± 4.4	12.6 ± 3.5	13.9 ± 9.8	15.1 ± 9.8
REM, %	20.2 ± 3.7	17.1 ± 3.8	23.6 ± 3.2^*∗*^	19.3 ± 2.4	21.7 ± 2.8	19.2 ± 4.9
SE, %	92.9 ± 5.5	92.5 ± 3.9	93.2 ± 3.1	96.7 ± 1.9	95.5 ± 2.1^*∗*^	89.7 ± 5.2

Notes. SL: Latency to onset of stage 1 sleep; Daytime: mean SL for 5 naps scheduled at 10:00, 12:00, 14:00, 16:00, and 18:00; Nighttime: mean SL for 4 naps at 23:00, 1:00, 3:00, and 5:00; Baseline: SL on the first 20-min interval of Baseline night sleep (light off at 23:00). See also Figure 1. Wake: wake time after sleep onset; sleep stages are given as percentage to Total Sleep Time (TST); SWS: slow wave sleep (stages 3 and 4 sleep); REM: Rapid Eye Movement sleep; SE: sleep efficiency expressed as percentage of sleep time in total bed time (i.e., TST plus total wake time). Level of significance for difference between patients and controls: ^*∗*^(*p* < 0.05).
